# Gut microbiota mediates the pro-pyroptosis effect of xierezhuyubuxu decoction in hepatocellular carcinoma

**DOI:** 10.3389/fmicb.2024.1481111

**Published:** 2025-02-19

**Authors:** Zhili Zeng, Mingxuan Feng, Fan He, Enxin Zhang, Xiushen Li, Zebiao Cao

**Affiliations:** ^1^State Key Laboratory of Traditional Chinese Medicine Syndrome, the Second Affiliated Hospital of Guangzhou University of Chinese Medicine, Guangzhou, Guangdong, China; ^2^Post-Doctoral Research Center, Guangdong Provincial Hospital of Chinese Medicine, Guangzhou, Guangdong, China; ^3^Guangdong Provincial Academy of Chinese Medical Sciences, Guangzhou, Guangdong, China; ^4^The Second Clinical College of Guangzhou University of Chinese Medicine, Guangzhou, Guangdong, China; ^5^School of Basic Medical Sciences, Guangzhou University of Chinese Medicine, Guangzhou, China; ^6^Department of Oncology, The First Affiliated Hospital of Guizhou University of Chinese Medicine, Guiyang, Guizhou, China; ^7^Department of Oncology, Shenzhen Bao’an Authentic TCM Therapy Hospital, Shenzhen, Guangdong, China; ^8^Department of Traditional Chinese Medicine, Jiangxi Maternal and Child Health Hospital, Nanchang, China

**Keywords:** xierezhuyubuxu decoction, hepatocellular carcinoma, pyroptosis, intestinal flora, fecal microbial transplantation (FMT)

## Abstract

**Introduction:**

Xierezhuyubuxu decoction (XRZYBXD) is prepared by adding and reducing the Dahuang Zhechong Pill, which is a traditional Chinese medicinal formula in “The Synopsis of Prescriptions of the Golden Chamber”. XRZYBXD has previously been reported to have good efficacy in treating Hepatocellular carcinoma (HCC) in clinical and basic research. However, its underlying mechanism in treating HCC has not been fully elucidated. The aim of the study is to investigate the pro-pyroptosis effect of XRZYBXD in HCC and the role of gut microbiota in this process.

**Methods:**

Firstly, we executed comprehensive analyses of XRZYBXD on pyroptosis, intestinal flora, microbial metabolites and intestinal barrier function using TUNEL, IHC, ELISA, WB, Q-PCR, 16S rRNA sequencing, and untargeted metabolomics in a H22 tumor-bearing mice model. Further, through rescue experiment of antibiotics-induced microbiota depletion and fecal microbial transplantation (FMT) experiment, the mechanism of XRZYBXD promoting pyroptosis of HCC by improving intestinal flora was verified.

**Results:**

We found that XRZYBXD medium and high dose significantly inhibited the growth of tumor and induced pyroptosis of hepatoma cells. They also modified intestinal ecological disorders by expansion of the abundance of beneficial bacteria (such as *Akkermansia muciniphila* and *Parabacteroides distasonis*) and reduction of the abundance of harmful bacteria (such as *Barnesiella intestinihominis*). Accordingly, microbiota metabolites and intestinal barrier function were also significantly improved by XRZYBXD.

**Discussion:**

Further, elimination of gut microbiota by antibiotics weakened the efficacy of XRZYBXD, and FMT with feces from the XRZYBXD high dose group achieved similar therapeutic efficacy as XRZYBXD. In brief, XRZYBXD promote pyroptosis of hepatoma cells via adjusting intestinal dysbiosis.

## 1 Introduction

Hepatocellular carcinoma (HCC) is a malignant tumor with high morbidity and mortality (Sung et al., 2021). Chronic liver inflammation and cirrhosis that driven by viral hepatitis, alcoholic liver disease and non-alcoholic fatty liver disease are crucial factors leading to the occurrence and progression of HCC. Although there are many treatments available, HCC patients still face the high recurrence and metastasis rate and low 5-year survival rate, suggesting that it is necessary to find more treatment strategy (Vogel et al., 2022).

Traditional Chinese Medicine (TCM) has been used to treat various diseases in China for more than 2,000 years. XRZYBXD is prepared by adding and reducing the Dahuang Zhechong Pill, a traditional Chinese medicinal formula in “The Synopsis of Prescriptions of the Golden Chamber”. XRZYBXD is composed of Dahuang (*Rheum palmatum L.*), Zhechong (*Eupolyphaga sinensis Walker*), Taoren (*Prunus davidiana (Carr.) Franch.*) and other Chinese medicine. Each herb has been checked with the latest revision of Medicinal Plant Names Services (MPNS) (MPNS, Royal Botanic Gardens, Kew, accessed on July 31, 2024).^1^ Previous clinical and experimental studies have shown that it has a good therapeutic effect on HCC (Dai et al., 2021; Liwei Liu et al., 2023; Chen et al., 2022) but its effect on pyroptosis has not been explored.

Pyrotosis is a new type of cell death mediated by gasdermin protein which promotes pore formation of cell membrane (Newton et al., 2024). Pyroptosis mainly relies on the inflammasome to activate some proteins of the caspase family, making it cut gasdermin protein and activate gasdermin protein. The activated gasdermin protein such as Gasdermin-D(GSDMD), transfer to the cell membrane to form pores, and result in cytoplasmic efflux, and eventually cell pyroptosis due to cell membrane rupture. There are classical and non-classical pathways in pyroptosis. Among them, the classical pathway is mainly related to caspase-1 and GSDMD, while the non-classical pathway is mainly associated with caspase-4, 5 or caspase-11 (Newton et al., 2024). Pyroptosis plays an important role in maintaining normal physiological function and morphology of tissues. Resistance to cell death is one of the hallmarks of cancer (Hanahan, 2022). Previous studies have shown that the occurrence and progression of gastric cancer, colon cancer and HCC are associated with resistance of cell pyroptosis (Wang et al., 2018; Wei et al., 2014; Williams et al., 2015).

Intestinal flora has also been recognized as one of the hallmarks of cancer (Hanahan, 2022), numerous studies have demonstrated that it plays a critical role in the maintenance of physiological homeostasis and the resistance of diseases, especially metabolic disorders and cancer (Jia et al., 2018; Liu et al., 2021). Balanced intestinal flora helps to prevent the occurrence of cancer, while intestinal dysbiosis may promote tumor formation and progression by disrupting intestinal barrier function and releasing carcinogenic metabolites (Schwabe and Jobin, 2013). Accordingly, targeting intestinal flora has been reported to be a promising therapeutic strategy for cancer treatment (Bu et al., 2020). Chinese herbal medicine in XRZYBXD, such as peach kernel, rhubarb and angelica, have been reported to have the effect of promoting bowel movement, and rhubarb, angelica, and American ginseng have the effect of regulating intestinal flora (Bu et al., 2020; Hu et al., 2022; Wang et al., 2016), but whether gut microbiota is one of the targets of XRZYBXD remains unknown.

Therefore, the present study is aim to explore the effect of XRZYBXD on pyroptosis of hepatoma cells and the role of gut microbiota in this process by integrating 16S rRNA sequencing, metabolomics, and microbial manipulation experiments. We found that XRZYBXD promoted pyroptosis of hepatoma cells via adjusting intestinal dysbiosis, which will contribute to clarify the anti-HCC mechanism of XRZYBXD and provide a scientific basis for developing novel drugs.

## 2 Materials and methods

### 2.1 XRZYBXD preparation and metabolites identification

The Chinese medicines in XRZYBXD prescription were purchased from the Second Affiliated Hospital of Guangzhou University of Traditional Chinese Medicine. XRZYBXD was prepared by combining two times of decocted Chinese medicine solution. Q-Orbitrap high resolution liquid-mass combination was applied to identify the metabolites of XRZYBXD.

### 2.2 Cell lines and culture

Mouse hepatoma cell H22 were purchased from China Typical Culture Storage Center (Wuhan, China); H22 cells were cultured in RPMI-1640 medium (Gibco) containing 10% FBS (fatal bovine serum) in cell incubator containing 5% CO_2_ at 37°C.

### 2.3 Animal

Male C57BL/6 mice aged at 8–9 weeks (18–20 g) were provided by Experimental Animal Center of Guangzhou University of Chinese Medicine. All animals were raised in the specific pathogen-free (SPF) animal house with light and darkness for 12 h each, and appropriate temperature and humidity. Mice have free access to food and purified water. Animal experiments were approved by the Animal Ethics Committee of Guangzhou Ling Fu Tuopu biological Co., LTD (Approval No. LFTOP-IACUC-2024-0130).

### 2.4 Subcutaneous tumor model

After 7 days of adaptive feeding, 5 mice were randomly chosen as the normal group (N), and 25 mice were used for building H22 tumor-bearing mice model. As we previously described (Zeng et al., 2023), the H22 cells (2 × 10^7^/mL) were inoculated subcutaneously into the right axilla of mice. After successfully modeling, the mice were randomly divided into 5 groups: tumor model group (T), XRZYBXD high dose (H), medium dose (M) and low dose (L) groups, and positive drug group (S). Mice in the XRZYBXD group were administered with a low dosage (13.77g/kg), medium dosage (27.54 g/kg) and high dosage (55.08 g/kg/day) via gavage, once a day. Tegafur, Gimeracil and Oteracil Potassium Capsules (S-1) (Approved by H20100135, Jiangsu, China) was used as the positive drug. The efficacy of S-1 is clear, and it is a commonly used clinical chemotherapy drug for the treatment of digestive system tumors. According to the body surface area of 1.6 m^2^ and the body weight of 60 kg for adults, the dosage of S-1 is 120 mg per day. According to the conversion formula of intragastric dose between mice and human, the intragastric dose of mice was 25 mg/kg, the intragastric volume was 0.1 mL/10 g, once a day. And the drug was configured to about 2.5 mg/mL. Mice in the normal group and tumor model group were given the same volume of saline. The body weight and tumor volume were measured every 3 days. The tumor volume was calculated by the formula V = (length × width^2^)/2. After 21 days of administration, mouse feces in model group and XRZYBXD high-dose group were collected to prepare for subsequent microbial manipulation experiments. Then the mice were anesthetized cervical vertebra dislocated and killed, and the blood of the eyeball, and the tumor, liver, kidney and ileum were taken out. The tumor, liver, kidney and ileum tissues were stained with HE, observed and photographed by light microscope (200×). Q-PCR (Quantitative real time polymerase chain reaction), and immunohistochemical (IHC) were performed using tumor and ileum tissues. Inflammatory factors including IL-1β, IL-18 in mice were assessed by ELISA (Enzyme-linked Immunosorbent Assay) kits.

### 2.5 16S rRNA sequencing

Fresh feces were collected in sterilized EP (Eppendorf) tubes and stored in the −80°C refrigerator for later use. Quality tests are performed on the feces first. Qualified DNA samples were selected and configured with the PCR reaction system for PCR amplification. Agencourt AMPure XP magnetic beads were used to purify the PCR amplification products and dissolved in the Elution Buffer. Then they were labeled and applied for library construction. The fragment range and concentration of the library were detected using Agilent 2100 Bioanalyzer. The qualified library was sequenced by HiSeq platform according to the size of the inserted fragment. Filter the data of offline, remaining high-quality clean data for later analysis. Reads are spliced into Tags by the relationship of overlap between reads. The Tags are clustered into OTU and compared with the database to annotate the species. Based on OTU and annotation results, sample species complexity analysis, inter-group species difference analysis, association analysis and function prediction were conducted.

### 2.6 Identification of fecal metabolites in mice

After 21 days of intervention, the feces of the tumor model group and the XRZYBXD high dose group were respectively tested for quality, and the qualified feces were detected for metabolites by LC-MS/MS (liquid chromatography tandem mass spectrometry). Bioinformatics analysis was used to screen differential metabolites.

### 2.7 Microbial manipulation experiments

A mixture of antibiotics (ABX) containing 1 mg/mL ampicillin, 1 mg/mL metronidazole, 1 mg/mL neomycin and 0.5 mg/mL vancomycin (Xu et al., 2020) was applied to build the pseudo-germ-free mouse model. The drinking water with ABX was changed three times a week. Further, in order to validate the anti-tumor and pro-pyroptosis effects of XRZYBXD-shaped intestinal flora, Mouse feces from the model group and the high-dose group of XRZYBXD of the first animal experiment were collected. Refer to previous literature (Zhong et al., 2022), 0.3g feces were added into 3 mL distilled water, thoroughly shaken and mixed, and impurities were filtered to make feces suspension, which was stored in the refrigerator at −80°C for future use. Pseudo-germ-free mice were administered with 200 μl fecal suspension by gavage for 21 days.

### 2.8 Q-PCR assay

Q-PCR assay was applied as we previously reported (Zeng et al., 2023). Mouse PCR primers were designed and purchased by Sangon Biotech (Shanghai, China), including NLRP3, Caspase 1, GSDMD, IL-1β, IL-18, occluding, claudin-1, ZO-1, Muc2 and the housekeeping gene primer ACTB. The primer sequences are shown in Table 1. The relative expression levels of each gene were obtained by analyzing and calculating 2^–ΔΔCt^.

**TABLE 1:** The primer sequences of murine.

Gene	Forward	Reverse
p53	ACCGCCGACCTATCCTTACCATC	GGCACAAACACGAACCTCAAAGC
Bcl-2	TGTAGAGAGGAGAACGCAGGTAGTG	GGCTTCTTCTTCTGTGTGGTGGTC
Bax	GCTACAGGGTTTCATCCAGGATCG	TGCTGTCCAGTTCATCTCCAATTCG
Caspase 9	GTGAAGAACGACCTGACTGCCAAG	GAGAGAGGATGACCACCACAAAGC
Caspase 3	CGTGTGCGAGATGAGGTGTTGG	CAGCAGCAACAGCAGACTACCG
ACTB	GGTTGTCTCCTGCGACTTCA	TGGTCCAGGGTTTCTTACTCC
NLRP3	GCTGCGATCAACAGGCGAGAC	CCATCCACTCTTCTTCAAGGCTGTC
Caspase 1	ATACAACCACTCGTACACGTCTTGC	TCCTCCAGCAGCAACTTCATTTCTC
GSDMD	ACTGAGGTCCACAGCCAAGAGG	GCCACTCGGAATGCCAGGATG
IL-1β	TCGCAGCAGCACATCAACAAGAG	AGGTCCACGGGAAAGACACAGG
IL-18	CAAAGTGCCAGTGAACCCCAGAC	ACAGAGAGGGTCACAGCCAGTC
Occludin	GGCGGCTATGGAGGCTATGG	CTAAGGAAGCGATGAAGCAGAAGG
Claudin-1	TGGGTTTCATCCTGGCTTCTCTG	TGGGTTTCATCCTGGCTTCTCTG
ZO-1	AAGCAGTGGAAGAAGTTACAGTTGAG	AGAAGGGCTGACGGGTAAATCC
Muc2	ACGCCTGTGACCTCTCAATCC	TGTGCTAGATGTTGCTGTGGTTAC

### 2.9 Western blot

Western blot assay was conducted as we previously reported (Zeng et al., 2023). The following antibodies were used in the Western blot assay including NLRP3 antibody [15101, Cell Signaling Technology (CST)], Cleaved caspase 1 antibody (89332, CST), GSDMD-N antibody (34667, CST), IL-1β antibody (12242, CST), IL-18 antibody (57058, CST), and β-actin antibody (4970, CST).

### 2.10 IHC assay

After paraffin section was dewaxed, citric acid antigen repair buffer was added for repair, sealed with 3%BSA at room temperature, then primary antibodies against occluding (27260-1-AP, Proteintech), claudin-1 (AF0127, Affinity) and ZO-1 (AF5145, Affinity) were added, and incubated at 4°C overnight. Next, secondary antibodies were used to incubate the slides for 50 min. DAB color solution was added for color development, and the time was controlled by microscope observation. When the color development turned to brownish yellow, it was terminated by washing with water and sealed with neutral gum, then observed and photographed under microscope and analyzed statistically. The total optical density (IOD) and area of each image were measured, and the average optical density (AOD) was calculated.

### 2.11 Statistics

The statistical software SPSS 25.0 was applied for statistical analyses. When the data satisfy both normal distribution and homogeneity of variance, one-way analysis of variance (ANOVA) was used. When the data do not meet the normal distribution or the variance is not uniform, the non-parametric Kruskal-Wallis H test was used. Statistical graphs were plotted by Graphpad Prism v.8.0 (Graphpad Software, USA). The statistical results were statistically different with *P* < 0.05.

## 3 Results

### 3.1 Identification of metabolites in XRZYBXD

A total of 417 metabolites were obtained in XRZYBXD based on Q-Orbitrap high resolution liquid-mass combination. Among them, 30 metabolites with high matching degree with mzCloud database were shown in Table 2 and Supplementary Figure 1.

**TABLE 2:** Chemical components of XRZYBXD based on Q-Orbitrap high resolution liquid mass spectrometry.

Peak no.	Components/Metabolite name	Formula	Molecular weight	RT [min]	Molecular Ion (m/z)
1	Uridine	C9 H12 N2 O6	244.06944	3.974	neg
2	Guanosine monophosphate (GMP)	C10 H14 N5 O8 P	363.05811	5.953	neg
3	Chlorogenic acid	C16 H18 O9	376.07702	8.341	neg
4	Neochlorogenic acid	C16 H18 O9	354.09488	8.343	neg
5	Rutin	C27 H30 O16	610.15295	12.967	neg
6	Afzelin	C21 H20 O10	432.10542	14.399	neg
7	Naringenin	C15 H12 O5	272.06857	14.648	neg
8	Nootkatone	C15 H22 O	218.16707	17.227	neg
9	Palmitic acid	C16 H32 O2	256.24010	22.851	neg
10	Catechin	C15 H14 O6	290.07884	9.125	pos
11	Berberine	C20 H17 N O4	335.11562	11.406	pos
12	Scopoletin	C10 H8 O4	192.04231	11.567	pos
13	Ferulic acid	C10 H10 O4	194.05755	11.936	pos
14	(-)-Caryophyllene oxide	C15 H24 O	220.18257	12.116	pos
15	Vitexin	C21 H20 O10	432.10550	12.222	pos
16	Quercetin	C15 H10 O7	302.04223	12.991	pos
17	Resveratrol	C14 H12 O3	228.07844	13.099	pos
18	Astragalin	C21 H20 O11	448.10015	13.698	pos
19	Isorhamnetin	C16 H12 O7	316.05800	13.745	pos
20	Salvianolic acid B	C36 H30 O16	718.15209	13.944	pos
21	Tiliroside	C30 H26 O13	594.13718	14.977	pos
22	Aloe-emodin	C15 H10 O5	270.05272	15.451	pos
23	Nobiletin	C21 H22 O8	402.13100	17.064	pos
24	Ginsenoside Rb1	C54 H92 O23	1130.58403	17.844	pos
25	Oleanolic acid	C30 H48 O3	438.34935	18.664	pos
26	Cryptotanshinone	C19 H20 O3	296.14092	18.959	pos
27	Ginsenoside Rd	C48 H82 O18	946.54912	19.425	pos
28	Tanshinone IIA	C19 H18 O3	294.12541	19.973	pos
29	Maltol	C6 H6 O3	126.02967	20.152	pos
30	Maslinic acid	C30 H48 O4	494.33708	20.966	pos

XRZYBXD, xierezhuyubuxu decoction; RT, retention time.

### 3.2 XRZYBXD inhibits hepatoma cells growth without body weight loss side effects

**FIGURE 1:**
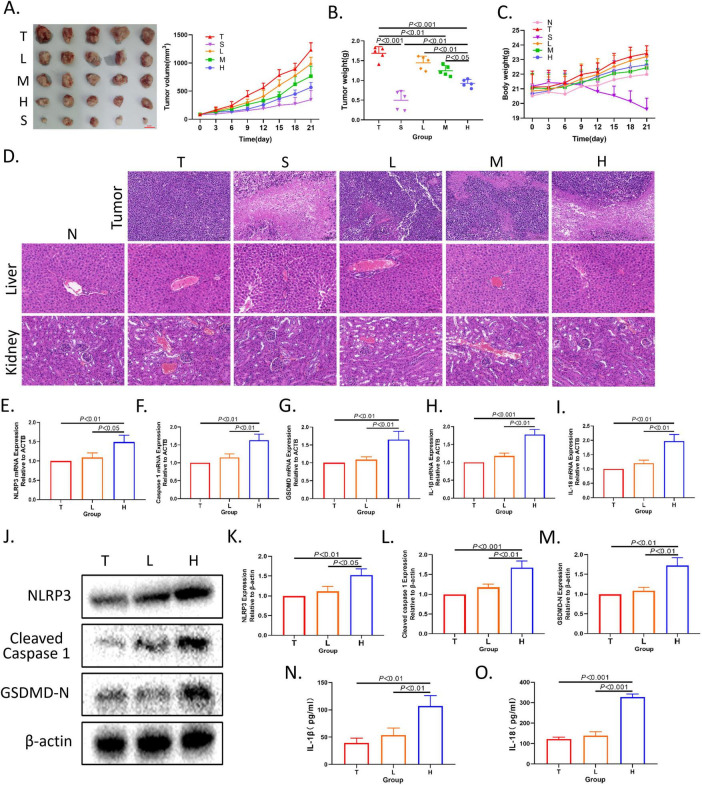
XRZYBXD inhibited tumor growth and promoted pyroptosis of hepatoma cells in H22 tumor-bearing mice model. **(A)** Tumor sizes and tumor growth curves after different intervention. **(B,C)** Tumor weight and body weight growth curves after different intervention. **(D)** HE staining of tumor, liver and kidney sections (Magnification × 200), the scale bar 100 μm. **(E–I)** The mRNA expressions of NLRP3, Caspase 1, GSDMD, IL-1β, and IL-18 by Q-PCR. **(J–M)** The protein expressions of NLRP3, Cleaved Caspase 1, and GSDMD using WB. **(N,O)** The levels of IL-1β and IL-18 in tumor tissue by ELISA. All data are presented as means ± SD (*n* = 5). XRZYBXD, xierezhuyubuxu decoction; Q-PCR, quantitative real time polymerase chain reaction; WB, western blot; ELISA, Enzyme-linked Immunosorbent Assay.

With different intervention, the growth trend of tumor volume in XRZYBXD high, medium dose group and S-1 group were significantly slower than that in tumor model group (Figure 1A). At the end of intervention, the tumor weight of XRZYBXD high, medium dose group and S-1 group were also significantly lower than that of the tumor model group (Figure 1B). Pathological examination showed that tumor cells in the three groups were loosely arranged, and their necrotic area of tumor tissue were significantly larger than that in the tumor model group (Figure 1D). Liver and kidney sections of mice suggested no significant abnormality in each group (Figure 1D). However, during the administration, compared with the other groups, the body weight of mice in the S-1 group showed a downward trend. At the end of intervention, the mice in S-1 group had the lowest body weight and were significantly lower than the other groups (Figure 1C). Therefore, it could be found that although the S-1 group had better anti-tumor effect, the mice lost weight more quickly, while XRZYBXD inhibited hepatoma cells growth without body weight loss side effects.

### 3.3 The pro-pyroptosis effect of XRZYBXD on hepatoma cells

As shown in Figures 1E–I, the mRNA relative expression level of NLRP3, Caspase 1, GSDMD, IL-1β and IL-18 in XRZYBXD high dose group were significantly higher than those in the tumor model group by Q-PCR. The protein expression level of NLRP3, caspase 1, GSDMD, IL-1β and IL-18 in XRZYBXD high dose group were also significantly higher than those in the tumor model group by WB (Figures 1J–M and Supplementary Figures 2F–H). Besides, Elisa results of tumor tissue displayed that compared with tumor model group, the levels of IL-1β and IL-18 in XRZYBXD high dose group were increased (Figures 1N, O). These results indicated that XRZYBXD may promote pyroptosis of hepatoma cells.

### 3.4 XRZYBXD restores community structure of intestinal flora

#### 3.4.1 Alpha diversity analysis

**FIGURE 2:**
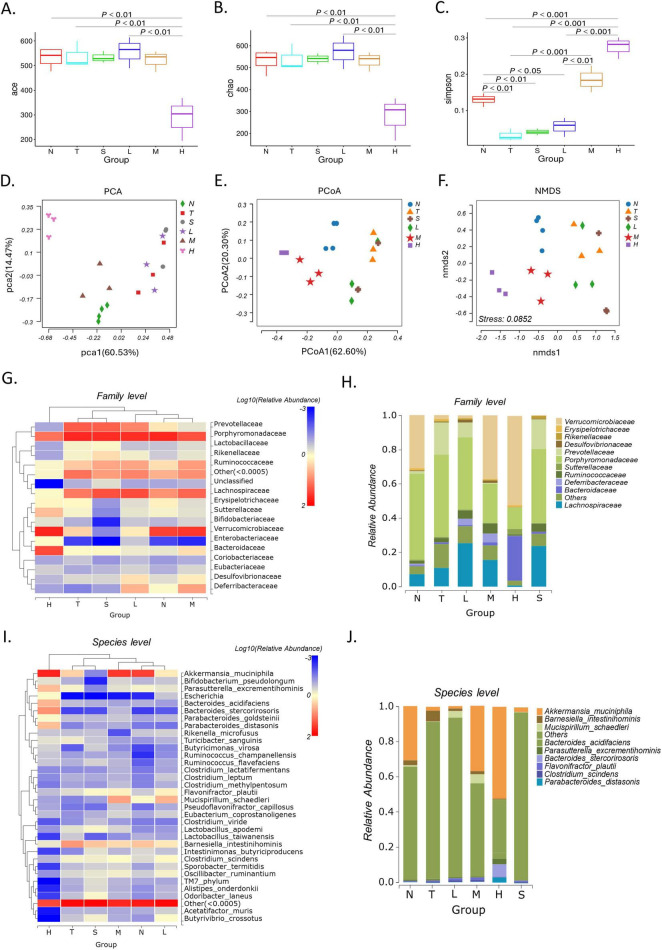
Effect of XRZYBXD on intestinal flora in H22 tumor-bearing mice model. **(A–C)** Alpha diversity analysis including ace, chao, and Simpson index. **(D–F)** Beta diversity analysis including PCA, PCoA, and NMDS. **(G,H)** Heatmap and stacking diagram illustrated the relative abundance of the top 17 and 11 bacterial communities at the family level, respectively. **(I,J)** Heatmap and stacking diagram showed the relative abundance of the top 33 and 9 bacterial communities at the species level, respectively. XRZYBXD, xierezhuyubuxu decoction; PCA, principal component analysis.

In order to reveal the underlying mechanism of the pro-pyroptosis effect of XRZYBXD in hepatoma cells, we analyzed intestinal flora by 16S rRNA sequencing. First, we explored the effects of XRZYBXD on the abundance and diversity of microbiota in mice. Alpha diversity analysis showed that compared with tumor model group, the ace and chao indices in the XRZYBXD high dose group decreased (Figures 2A, B). In addition, compared with normal group, the simpson indices in the tumor model group decreased, but prominently increased after XRZYBXD intervention (Figure 2C). These results indicated that XRZYBXD reversed the reduction in diversity of bacterial community induced by hepatoma.

#### 3.4.2 Beta diversity analysis

Beta diversity with Principal component analysis (PCA), PCoA and NMDS was applied to explore the changes in gut microbiota community among different interventions (Figures 2D–F). Both results of PCA and PCoA showed that there was remarkable difference between the normal group and tumor model group, and the structure of intestinal flora was significantly changed after XRZYBXD treatment compared with tumor model group (Figures 2D, E). Besides, beta diversity of NMDS further confirmed the results obtained from PCA and PCoA, the stress value of 0.0852 < 0.1 indicated good reliability of our findings (Figure 2F).

#### 3.4.3 Species analysis

**FIGURE 3:**
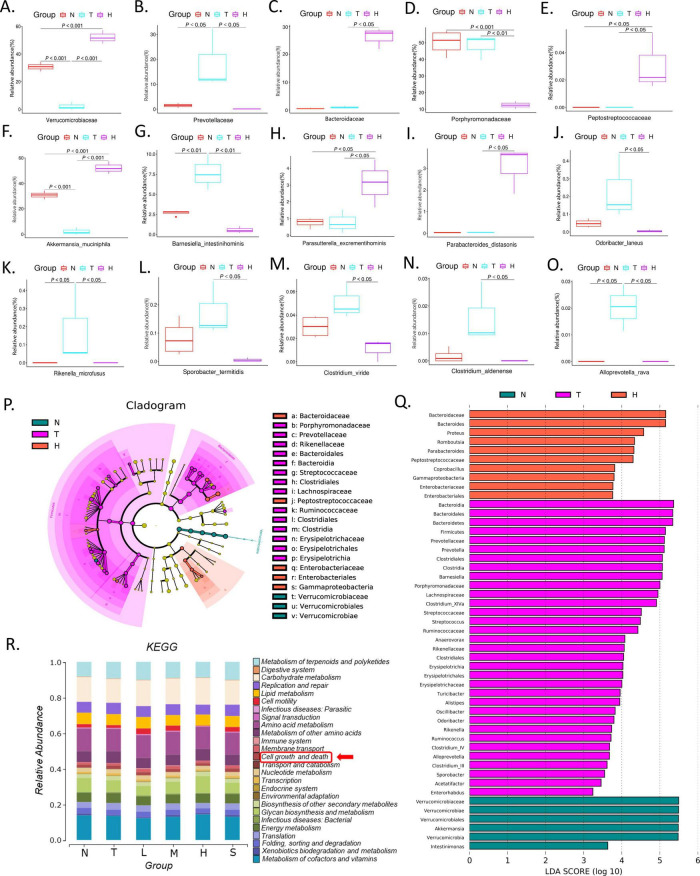
Effect of XRZYBXD on relative abundance of intestinal flora in H22 tumor-bearing mice model. **(A–O)** 5 differential bacterial family and 10 differential bacterial species, respectively. **(P,Q)** LefSe analysis of different groups with LDA score (log10) > 3, Left: LefSe evolutionary branching diagram; Right: LDA histogram; XRZYBXD, xierezhuyubuxu decoction. **(R)** KEGG enrichment analysis of gut microbiota. KEGG, Kyoto Encyclopedia of Genes and Genomes.

We further investigated the change of the relative abundance of gut microbiota under different intervention. At the family level, the relative abundance of *Verrucomicrobiaceae*, *Prevotellaceae*, *Bacteroidaceae*, *Porphyromonadaceae* and *Peptostreptococcaceae* changed significantly after different treatment (Figures 2G, H). Compared with normal group, there was a decrease of 28.6% in *Verrucomicrobiaceae* and an increase of 17.1% in *Prevotellaceae* in the tumor model group, but XRZYBXD intervention significantly reversed these changes (Figure 2H). Besides, the relative abundance of *Bacteroidaceae* and *Peptostreptococcaceae* increased, while *Porphyromonadaceae* prominently decreased after XRZYBXD intervention (Figures 3C–E).

At the species level, the top 33 microbiota were visualized using heatmap and the top 9 were shown in corresponding stacked diagram (Figures 2I, J). As can be seen in the diagram, the dominant bacterial communities in normal group were *Akkermansia muciniphila*, *Barnesiella intestinihominis*. After modeling, the relative abundance of *Akkermansia muciniphila* decreased and *Barnesiella intestinihominis* increased significantly, but they were both reversed after XRZYBXD treatment (Figures 2J, 3F, G). Besides, the relative abundance of *Parasutterella excrementihominis*, *Parabacteroides distasonis* increased, while *Odoribacter laneus*, *Rikenella microfusus*, *Sporobacter termitidis*, *Clostridium viride*, *Clostridium aldenense*, *Alloprevotella rava* decreased prominently after XRZYBXD intervention (Figures 3H–O).

#### 3.4.4 LefSe analysis and KEGG pathway enrichment

In LefSe analysis, a total of 49 biomarkers with LDA score > 3 were screened. At family level, LEfSe analysis revealed that *Verrucomicrobiaceae* were biomarkers of normal group, *Prevotellaceae*, *Porphyromonadaceae*, *Lachnospiraceae*, *Streptococcaceae*, *Ruminococcaceae*, *Rikenellaceae*, and *Erysipelotrichaceae* were biomarkers of tumor model group, *Enterobacteriaceae*, *Peptostreptococcaceae*, *Bacteroidaceae*, *Porphyromonadaceae* were biomarkers of XRZYBXD high dose group (Figures 3P, Q). Further, Kyoto Encyclopedia of Genes and Genomes (KEGG) enrichment analysis showed that intestinal flora was enriched in pathways associated with cell growth and death (Figure 3R).

### 3.5 XRZYBXD strengthens intestinal barrier

**FIGURE 4:**
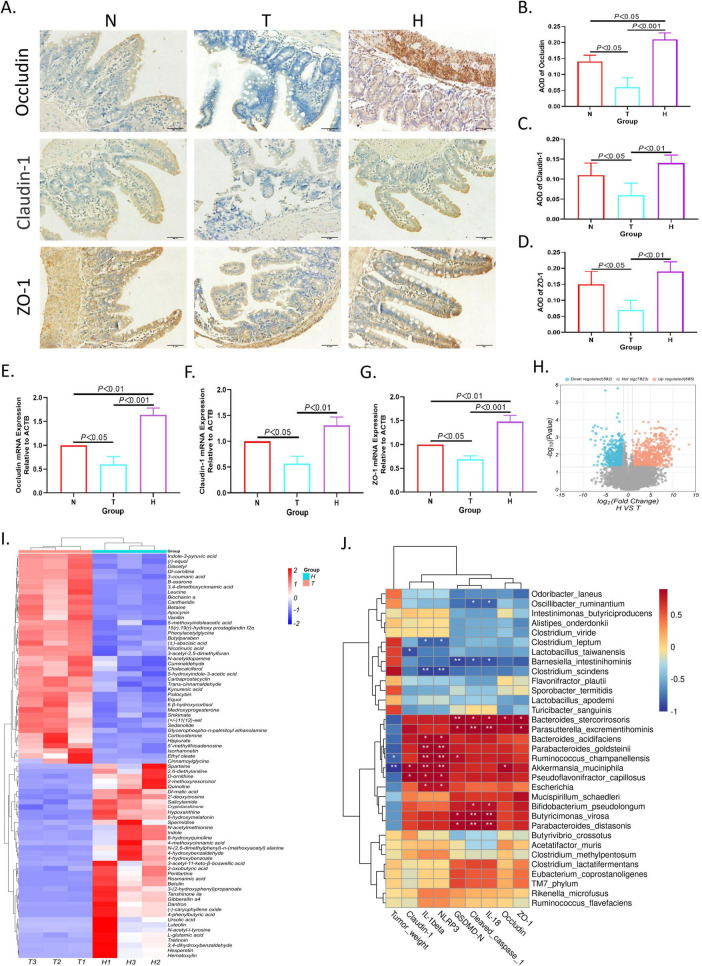
Effect of XRZYBXD on intestinal barrier and metabolites in H22 tumor-bearing mice model. **(A–D)** The protein expressions of occluding, claudin-1 and ZO-1 in ileal tissues by IHC assay (Magnification × 200), the scale bar represents 50 μm. **(E–G)** The mRNA expressions of occluding, claudin-1 and ZO-1 using Q-PCR. **(H)** The differential metabolites between tumor model and XRZYBXD high dose group were presented by volcano maps. **(I)** 79 differential metabolites between tumor model and XRZYBXD high dose group after matching with database were presented by heatmap. **(J)** Correlation analysis between top 33 bacterial species and tumor weight, NLRP3, cleaved caspase 1, GSDMD, IL-1β, IL-18, occluding, claudin-1 and ZO-1. XRZYBXD, xierezhuyubuxu decoction; Q-PCR, quantitative real time polymerase chain reaction; IHC, immunohistochemical; GSDMD, gasdermin.

H&E staining, IHC and Q-PCR were used to investigate the effect of XRZYBXD on intestinal barrier. As we can see from the normal group in H&E staining (Supplementary Figure 2A), the structure of each layer of ileum was clear, the villi were intact, the mucosal epithelial cells were densely arranged, and no obvious pathological variations can be observed. However, in the tumor model group, the ileum structure was disturbed, the villi were broken, and the goblet cells were reduced, the mucosal epithelium of the ileum was necrotic and exfoliated. After treatment of XRZYBXD high dose, the pathological changes of the ileum structure, mucosa and goblet cells were restored (Figure 4A). Further, occluding, claudin-1, ZO-1 and mucin 2(Muc2) were applied to assess intestinal barrier function under different invention. Compared with normal group, the expression of occluding, claudin-1, ZO-1 and Muc2 in tumor model group were prominently reduced, but they were all restored after the intervention of XRZYBXD (Figures 4B–G and Supplementary Figure 2B).

### 3.6 XRZYBXD improves microbial metabolites

We further investigated the metabolites produced by gut flora using feces through untargeted metabolomics. Based on the threshold of fold change (FC) > 2 and *P*-value < 0.05, compared with tumor model group, we identified 592 down-regulated and 685 up-regulated metabolites as shown in volcano plot (Figure 4H). Further, we identified 79 differential metabolites whose retention time matched with BGI metabolites database (BMDM) or mzCloud database and presented the results using a heatmap and table (Figure 4I and Table 3). Among these differential metabolites, compared with the tumor model group, differential metabolites whose relative content increased following XRZYBXD intervention such as 8-hydroxyquinoline, Indole, N-(2,6-dimethylphenyl)-n-(methoxyacetyl) alanine, N-acetylmethionine, 2-methoxyresorcinol, D-ornithine, Quinoline, Ursolic acid, Dl-malic acid and Spermidine, while Betaine, Cholecalciferol, Dl-carnitine, Trans-cinnamaldehyde, 3-coumaric acid, 5-hydroxyindole-3-acetic acid, Carbaprostacyclin, Cuminaldehyde, Ethyl oleate, N-acetyldopamine, B -asarone and so on decreased prominently following XRZYBXD intervention.

**TABLE 3:** Metabolites in feces of XRZYBXD group compared with model group based on LC-MS/MS.

No.	Components/Metabolite name	Formula	Molecular weight	RT [min]	Molecular Ion
1	Gibberellin a4	C19 H24 O5	332.1619	4.954	neg
2	Hematoxylin	C16 H14 O6	302.0786	4.356	neg
3	Luteolin	C15 H10 O6	286.0473	6.953	neg
4	Rosmarinic acid	C18 H16 O8	360.0841	3.237	neg
5	3-(2-hydroxyphenyl) propanoate	C9 H10 O3	166.0629	3.745	neg
6	3-coumaric acid	C9 H8 O3	164.0473	1.726	neg
7	Equol	C15 H14 O3	242.094	6.653	neg
8	5-hydroxyindole-3-acetic acid	C10 H9 N O3	191.0582	2.981	neg
9	Kynurenic acid	C10 H7 N O3	189.0425	1.357	neg
10	Shikimate	C7 H10 O5	174.0526	0.709	neg
11	N-acetyl-l-tyrosine	C11 H13 N O4	223.0841	2.733	neg
12	L-glutamic acid	C5 H9 N O4	147.053	0.638	neg
13	2-oxobutyric acid	C4 H6 O3	102.0317	0.624	neg
14	Nicotinuric acid	C8 H8 N2 O3	180.0534	3.025	neg
15	Cinnamoylglycine	C11 H11 N O3	205.0737	4.494	neg
16	Ursolic acid	C30 H48 O3	456.3595	9.949	neg
17	Tretinoin	C20 H28 O2	300.2085	8.950	neg
18	Medroxyprogesterone	C22 H32 O3	344.2351	8.893	neg
19	Psilocybin	C12 H17 N2 O4 P	284.0926	3.643	neg
20	Dantron	C14 H8 O4	240.0418	6.371	neg
21	Butylparaben	C11 H14 O3	194.0941	7.365	neg
22	Hippurate	C9 H9 N O3	179.0583	2.923	neg
23	Hypoxanthine	C5 H4 N4 O	136.0385	0.723	neg
24	Dl-malic acid	C4 H6 O5	134.0215	0.616	neg
25	Glycerophospho-n-palmitoyl ethanolamine	C21 H44 N O7 P	453.2848	9.542	neg
26	(±)11(12)-eet	C20 H32 O3	320.2348	8.999	neg
27	15(r),19(r)-hydroxy prostaglandin f2α	C20 H34 O6	370.2351	5.457	neg
28	Betulin	C30 H50 O2	442.3812	9.829	pos
29	Tanshinone iia	C19 H18 O3	294.1255	8.956	pos
30	Sparteine	C15 H26 N2	234.2097	6.138	pos
31	(-)-caryophyllene oxide	C15 H24 O	220.1828	8.523	pos
32	3-acetyl-11-keto-β-boswellic acid	C32 H48 O5	512.3484	8.968	pos
33	Perillartine	C10 H15 N O	165.1158	3.612	pos
34	Hesperetin	C16 H14 O6	302.079	5.581	pos
35	2,6-diethylaniline	C10 H15 N	149.1207	6.418	pos
36	N-(2,6-dimethylphenyl)-n- (methoxyacetyl) alanine	C14 H19 N O4	265.1314	4.234	pos
37	4-methoxycinnamic acid	C10 H10 O3	178.0633	4.721	pos
38	4-hydroxybenzoate	C7 H6 O3	138.0321	3.576	pos
39	8-hydroxyquinoline	C9 H7 N O	145.053	4.984	pos
40	3,4-dihydroxybenzaldehyde	C7 H6 O3	138.0321	3.411	pos
41	2-methoxyresorcinol	C7 H8 O3	140.0476	4.716	pos
42	Indole	C8 H7 N	117.058	4.984	pos
43	6-hydroxymelatonin	C13 H16 N2 O3	248.1161	3.911	pos
44	N-acetylmethionine	C7 H13 N O3 S	191.062	3.594	pos
45	4-phenylbutyric acid	C10 H12 O2	164.084	9.049	pos
46	D-ornithine	C5 H12 N2 O2	132.09	0.611	pos
47	4-hydroxybenzaldehyde	C7 H6 O2	122.0369	4.004	pos
48	Spermidine	C7 H19 N3	145.1581	0.557	pos
49	Cryptotanshinone	C19 H20 O3	296.1412	8.569	pos
50	2′-deoxyinosine	C10 H12 N4 O4	252.086	1.238	pos
51	Quinoline	C9 H7 N	129.0581	5.837	pos
52	Salicylamide	C7 H7 N O2	137.0478	1.312	pos
53	3-acetyl-2,5-dimethylfuran	C8 H10 O2	138.0683	5.061	pos
54	Sedanolide	C12 H18 O2	194.131	5.070	pos
55	Trans-cinnamaldehyde	C9 H8 O	132.0577	5.343	pos
56	5-methoxyindoleacetic acid	C11 H11 N O3	205.0742	6.721	pos
57	Cuminaldehyde	C10 H12 O	148.0891	7.905	pos
58	Indole-3-pyruvic acid	C11 H9 N O3	203.0587	3.568	pos
59	5′-methylthioadenosine	C11 H15 N5 O3 S	297.0895	3.498	pos
60	Cholecalciferol	C27 H44 O	384.3394	9.500	pos
61	N-acetyldopamine	C10 H13 N O3	195.0899	2.781	pos
62	(r)-equol	C15 H14 O3	242.0943	6.193	pos
63	Isorhamnetin	C16 H12 O7	316.0582	6.223	pos
64	(±)-abscisic acid	C15 H20 O4	264.1362	5.674	pos
65	Diacetyl	C4 H6 O2	172.0739	3.250	pos
66	Biochanin a	C16 H12 O5	284.0684	5.910	pos
67	Carbaprostacyclin	C21 H34 O4	350.2456	7.337	pos
68	6 β-hydroxycortisol	C21 H30 O6	378.2042	5.901	pos
69	Dl-carnitine	C7 H15 N O3	161.1052	0.735	pos
70	Cantharidin	C10 H12 O4	196.0739	4.147	pos
71	Vanillin	C8 H8 O3	152.0476	4.720	pos
72	Apocynin	C9 H10 O3	166.0634	5.869	pos
73	B -asarone	C12 H16 O3	208.1103	6.264	pos
74	Phenylacetylglycine	C10 H11 N O3	193.0743	4.263	pos
75	Leucine	C6 H13 N O2	131.0948	0.670	pos
76	Corticosterone	C21 H30 O4	346.2143	6.249	pos
77	3,4-dimethoxycinnamic acid	C11 H12 O4	208.074	3.460	pos
78	Betaine	C5 H11 N O2	117.0789	0.706	pos
79	Ethyl oleate	C20 H38 O2	310.2869	9.683	pos

XRZYBXD, xierezhuyubuxu decoction; LC-MS/MS, liquid chromatography tandem mass spectrometry; RT, retention time.

### 3.7 Correlation analysis

Spearman correlation analysis was conducted to investigate the association between gut microbiota and tumor weight, as well as indicators of hepatoma cell pyroptosis and intestinal barrier function. We found that probiotic such as *Akkermansia muciniphila*, *Parabacteroides distasonis*, and *Parasutterella excrementihominis* were positively correlated with indicators of pyroptosis, while harmful bacteria such as *Barnesiella intestinihominis*, *Clostridium scindens* showed negative correlation with these indicators. However, only *Akkermansia muciniphila* had strong negative correlation with tumor weight (Figure 4J). Additionally, many microfloras such as *Akkermansia muciniphila*, *Parasutterella excrementihominis*, *Barnesiella intestinihominis* and *Bacteroides_stercorirosoris* also had close association with indicators of intestinal barrier function.

**FIGURE 5:**
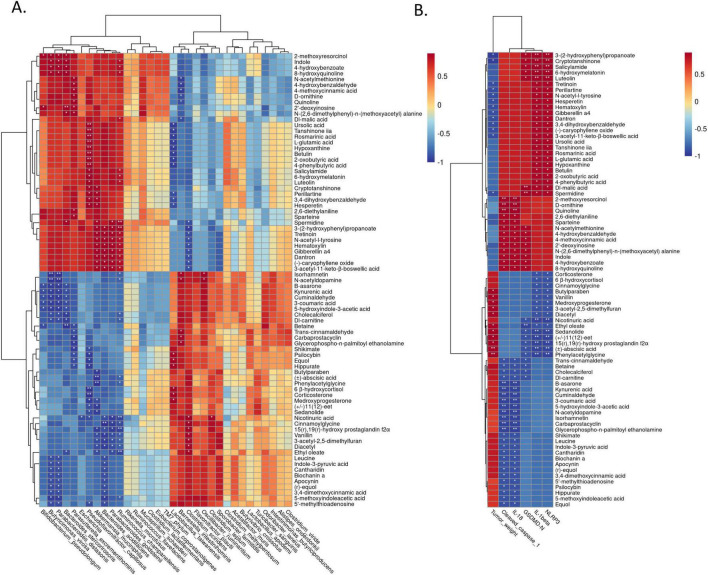
Correlation analysis of differential metabolites with gut microbiota and indicators. **(A)** Correlation analysis between differential metabolites and top 33 bacterial species; **(B)** correlation analysis between differential metabolites and tumor weight, NLRP3, cleaved caspase 1, GSDMD, IL-1β, IL-18. GSDMD, Gasdermin.

Then, we further explored the association between microflora and metabolites through spearman correlation analysis. The most important microflora *Akkermansia muciniphila* exhibited significant positive correlations with several differential metabolites including 3-(2-hydroxyphenyl) propanoate, Cryptotanshinone, Dantron, Gibberellin a4, Hematoxylin, N-acetyl-l-tyrosine, Tretinoin, Spermidine, (-)-caryophyllene oxide, 3,4-dihydroxybenzaldehyde, 3-acetyl-11-keto-β-boswellic acid, Perillartine and Hesperetin, while *Akkermansia muciniphila* showed prominent negative correlations with several differential metabolites including (±)-abscisic acid, Phenylacetylglycine, (±)11(12)-eet, 15(r),19(r)-hydroxy prostaglandin f2α, Butylparaben, Sedanolide, Medroxyprogesterone, Ethyl oleate, 3-acetyl-2,5-dimethylfuran, Diacetyl and Vanillin (Figure 5A).

Interestingly, as we can see from Figure 5B, when spearman correlation analysis was applied to investigate the relationship between differential metabolites and tumor weight, as well as indicators of pyroptosis, we found that the above-mentioned differential metabolites who had significant correlation with *Akkermansia muciniphila*, also had significant correlation with tumor weight and indicators of pyroptosis. These indicated that microfora especially *Akkermansia muciniphila* may have anti-tumor effect through their metabolites (Figure 5B).

### 3.8 Intestinal flora mediates the pro-pyroptosis effect of XRZYBXD

**FIGURE 6:**
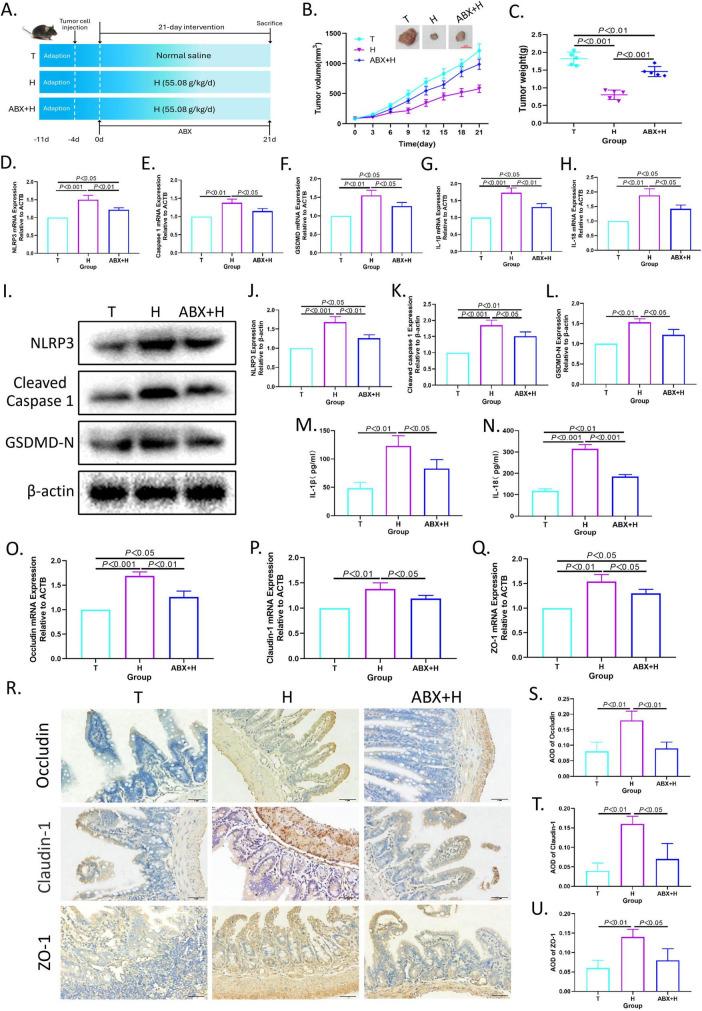
The depletion of intestinal flora weakened the efficacy of XRZYBXD. **(A)** The animal experimental design (*n* = 5). ABX was applied to deplete intestinal flora in ABX + H group through drinking water containing ABX for 21 days. **(B)** Tumor sizes and tumor growth curves after different intervention. **(C)** Tumor weight after different intervention. **(D–H)** The mRNA expressions of NLRP3, Caspase 1, GSDMD, IL-1β and IL-18 in tumor tissues using Q-PCR. **(I–L)** The protein expressions of NLRP3, Cleaved Caspase 1 and GSDMD in tumor tissues by WB. **(M,N)** The levels of IL-1β and IL-18 in tumor tissue by ELISA. **(O–Q)** The mRNA expressions of occluding, claudin-1 and ZO-1 based on Q-PCR. **(R–U)** The protein expressions of occluding, claudin-1 and ZO-1 in intestinal tissues based on IHC assay (Magnification × 200), the scale bar represents 50 μm. All data are presented as means ± SD (*n* = 5). XRZYBXD, xierezhuyubuxu decoction; ABX, antibiotics; GSDMD, gasdermin; Q-PCR, quantitative real time polymerase chain reaction; WB, western blot; ELISA, Enzyme-linked Immunosorbent Assay; IHC, immunohistochemical.

As shown in Figure 6A, to verify the contribution of intestinal flora in the efficacy of XRZYBXD, ABX was used for eliminating intestinal flora in H22 tumor-bearing mice during XRZYBXD treatment. Intriguingly, the tumor volume and tumor weight of ABX + XRZYBXD high dose group were higher than that of XRZYBXD high dose group, but lower than that of tumor model group (Figures 6B, C), which indicated that the elimination of gut microbiota weakened the anti-tumor effect of XRZYBXD. In addition, compared with XRZYBXD high dose group, the mRNA and protein expression of NLRP3, caspase 1, GSDMD, IL-18 and IL-1β were markedly decreased in ABX + XRZYBXD high dose group, which suggested that the pro-pyroptosis effect of XRZYBXD in hepatoma cells was attenuated by the depletion of intestinal flora (Figures 6D–N and Supplementary Figures 2I–K). Additionally, through H&E staining, we found that although the damage degree of ileum in ABX + XRZYBXD high dose group mice was lower than that in model group, there were still some villi morphology disturbed, goblet cells reduced, and some epithelial cells shed (Supplementary Figure 2A). Following the depletion of microbiota, the expression of occluding, claudin-1, ZO-1 and Muc2 also had a noticeable decrease when compared with XRZYBXD high dose group (Figures 6O–U and Supplementary Figure 2C).

**FIGURE 7:**
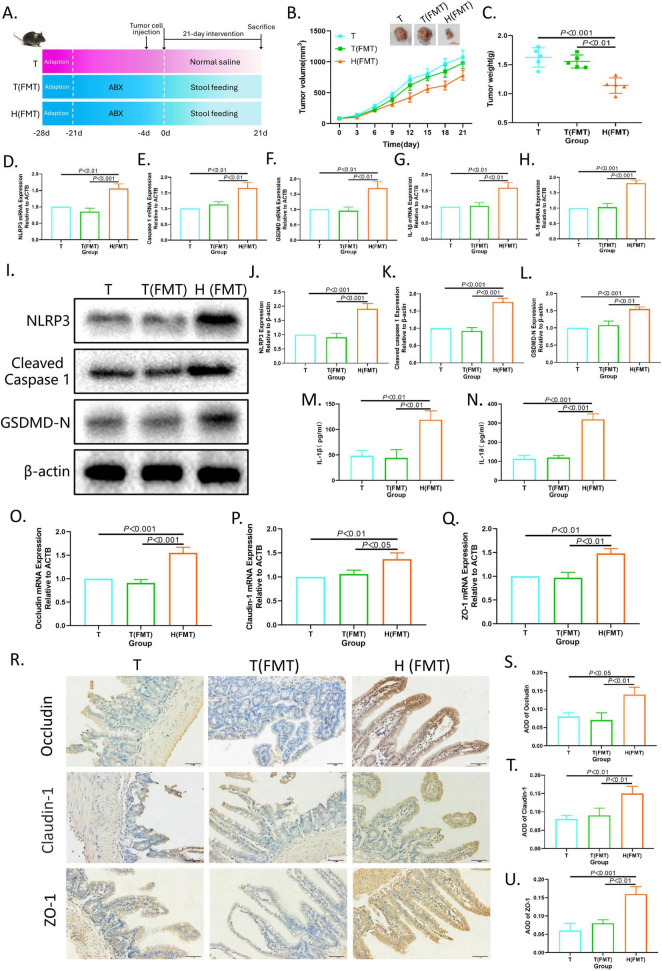
Intestinal flora mediates the pro-pyroptosis effect of XRZYBXD on hepatoma cells. **(A)** Schematic diagram of experimental design (*n* = 5). Fecal microbiota from the tumor model group were transplanted into T(FMT) group by gavage, while fecal microbiota from the XRZYBXD high dose group were transplanted into H(FMT) group via gavage. **(B)** Tumor sizes and tumor growth curves after different intervention. **(C)** Tumor weight after different intervention. **(D–H)** The mRNA expressions of NLRP3, Caspase 1, GSDMD, IL-1β and IL-18 in tumor tissues using Q-PCR. **(I–L)** The protein expressions of NLRP3, Cleaved Caspase 1 and GSDMD in tumor tissues based on WB. **(M,N)** The levels of IL-1β and IL-18 in tumor tissue by ELISA. **(O–Q)** The mRNA expressions of occluding, claudin-1 and ZO-1 based on Q-PCR. **(R–U)** The protein expressions of occluding, claudin-1 and ZO-1 in intestinal tissues based on IHC assay (Magnification × 200), the scale bar represents 50 μm. All data are presented as means ± SD (*n* = 5). XRZYBXD, xierezhuyubuxu decoction; ABX, antibiotics; FMT, fecal microbiota transplantation; GSDMD, gasdermin; Q-PCR, quantitative real time polymerase chain reaction; WB, western blot; ELISA, Enzyme-linked Immunosorbent Assay; IHC, immunohistochemical.

Furthermore, fecal microbiota from XRZYBXD high dose group and tumor model group were respectively transplanted into pseudo-sterile H22 tumor-bearing mice to confirm the role of microbiota in the pro-pyroptosis effect of XRZYBXD (Figure 7A). Compared with mice that were transplanted with fecal microbiota from the tumor model group (T(FMT) group), mice that were transplanted with fecal microbiota from the XRZYBXD high dose group (H(FMT) group) had significant decrease in tumor volume and weight, but there was no significant difference between T (FMT) group and tumor model group (Figures 7B, C). Meanwhile, the mRNA and protein expression levels of NLRP3, caspase 1, GSDMD, IL-1β and IL-18 in H(FMT) group were higher than that in T(FMT) group, while there were no noticeable difference between T(FMT) group and tumor model group (Figures 7D–N). Besides, H&E staining showed that the destroyed of FMT(T) group was similar to model group, but significantly restored after the intervention of FMT(H) (Supplementary Figure 2A). Meanwhile, the expression of occluding, claudin-1, ZO-1 and Muc2 were also increased following H(FMT) treatment (Figures 7O–U and Supplementary Figure 2D). These data suggested that the FMT experiment was successfully executed and XRZYBXD-shaped intestinal flora had a prominent promoting effect on hepatoma cell pyroptosis.

## 4 Discussion

Our previous studies have proven that XRZYBXD has a good anti-hepatoma effect by accelerating apoptosis of hepatoma cells (Zeng et al., 2023), but its underlying mechanism has not been fully elucidated.

Resistance to cell death is regarded as one of the hallmarks of cancer (Hanahan, 2022). Pyroptosis is a new type of programmed cell death mediated by gasdermin (GSDMD). The NLRP3 inflammasome is a key link in the initiation of pyroptosis. The inflammasome senses the external stimulus signal, recruits and activates caspase-1, caspase-1 cuts and activates IL-18, IL-1β and other inflammatory factors, at the same time, caspase-1 cuts the N-terminal sequence of GSDMD, and makes it bind to the cell membrane to produce membrane pores, resulting in cell pyroptosis. In present study, compared with tumor model group, the expression of NLRP3, caspase 1, GSDMD and the level of IL-1β and IL-18 in XRZYBXD high dose group were increased. The results indicated that XRZYBXD may also promote pyroptosis of hepatoma cells.

XRZYBXD has the effect of promote intestinal motility, besides, we found that many metabolites in XRZYBXD, including Resveratrol (Che et al., 2020), Vitexin (Li S. et al., 2021), Quercetin (Hisaka et al., 2020; Shabbir et al., 2021), Salvianolic acid B (Bai et al., 2021), Ginsenoside Rb1 (Bai et al., 2021), Ginsenoside Rd (Jin et al., 2020), Berberine (Yang L. et al., 2021) not only have anti-tumor effect, but also can regulate intestinal flora. Thus, we further investigate the role of gut microbiota in the anti-hepatoma effect of XRZYBXD.

Similar to previous evidence (Zhang et al., 2021), our results showed that the diversity of microflora was differentially decreased in model group, but they were recovered by XRZYBXD treatment. Beta diversity analysis also showed that there were significant structural differences between the normal group and tumor model group, as well as tumor model group and XRZYBXD group. Interestingly, KEGG enrichment analysis showed that intestinal flora was noticeably enriched in pathways associated with cell growth and death, so we further investigate the correlation between microbiota and hepatoma cell death. Spearman correlation analysis indicated that probiotic such as *Akkermansia muciniphila*, *Parabacteroides distasonis*, and *Parasutterella excrementihominis* were positively correlated with indicators of pyroptosis, while harmful bacteria such as *Barnesiella intestinihominis*, *Clostridium_scindens* showed negative correlation with these indicators. However, only *Akkermansia muciniphila* had strong negative correlation with tumor weight.

At the species level, we found that compared with the tumor model group, the abundance of probiotic such as *Akkermansia muciniphila* (Schneider et al., 2022) and *Parabacteroides distasonis* (Koh et al., 2018) were significantly increased after XRZYBXD treatment, while conditional pathogens such as *Escherichia* (Liu et al., 2019) and *Barnesiella intestinihominis* (Touchefeu et al., 2021) were decreased by XRZYBXD (Figures 3I, J). *Akkermansia muciniphila*, a well-known probiotic, was a dominant species after high dose XRZYBXD invention, it can be seen that, *Akkermansia muciniphila may play an important role in* the anti-tumor effect of XRZYBXD. *Akkermansia muciniphila* can improve host metabolic disorders, decrease lipogenesis, gluconeogenesis, metabolic endotoxemia and insulin resistance, reduce body weight, and regulate inflammatory immune response (Aggarwal et al., 2022; Rodrigues et al., 2022; Sanjiwani et al., 2022). Additionally, it has shown potential for regulating intestinal homeostasis, restoring intestinal barrier function and reducing liver inflammation and fibrosis (Schneider et al., 2022). Accumulating evidences suggest that *Akkermansia muciniphila* is a promising therapeutic target, marker and therapeutic agent for metabolic syndrome and related diseases (Aggarwal et al., 2022; Zhou and Zhang, 2019). However, current reports on *Akkermansia muciniphila* mostly focus on metabolic syndrome, type 2 diabetes, and inflammatory bowel disease, its role in liver cancer has not been reported. This study is the first to report that decreased abundance of *Akkermansia muciniphila* may be associated with the progression of HCC. Accordingly, the recovery of *Akkermansia muciniphila* in abundance may be an important factor for the efficacy of XRZYBXD.

*Akkermansia muciniphila* is belonging to the family *Verrucomicrobiaceae* (Cani et al., 2022). In our study, at the family level, we observed that the relative abundance of *Verrucomicrobiaceae* was significantly reduced after molding, but increased following XRZYBXD intervention. It has been reported that the decline in the abundance of *Verrucomicrobiaceae* is related to the development of schizophrenia (Zeng et al., 2024) and colitis (Li et al., 2022). However, the relationship between *Verrucomicrobiaceae* and the occurrence and development of HCC has not been reported, our study is the first to report that *Verrucomicrobiaceae* may be associated with the progression of HCC. In addition, *Bacteroidaceae* has been reported to have negative correlation with the progression of liver cancer (Ma et al., 2023). In our study, *Bacteroidaceae* increased significantly after XRZYBXD treatment. Additionally, *Prevotellaceae* was reported to be associated with oral cancer (Xiang et al., 2024) and microvascular invasion of liver cancer (Zhang et al., 2022). Jiang et al. (2020) reported that with the progression of liver cancer, the abundance of *Porphyromonadaceae* gradually increased. In our study, the relative abundance of *Prevotellaceae* and *Porphyromonadaceae* decreased significantly after XRZYBXD treatment. These results suggested that XRZYBXD ameliorated gut microbiota through increasing probiotics and decreasing harmful bacteria.

Next, we used untargeted metabolomics to explore the effects of XRZYBXD on microbial metabolites. Corresponding to changes in the structure of the flora, the composition of microbial metabolites changed significantly after XRZYBXD intervention. Then, we further explored the association between microflora and metabolites through spearman correlation analysis. The most important microflora *Akkermansia muciniphila* exhibited significant positive correlation with several differential metabolites. Among these differential metabolites, Cryptotanshinone (Zhao et al., 2019), Spermidine (Wang et al., 2017) 和 Hesperetin (Zhang et al., 2015) have been reported to have effect on inhibiting progression of liver cancer; Dantron can inhibit the growth and migration of Hela cell (Chou et al., 2018), and may enhance the sensitivity of pancreatic cancer cells to adriamycin (Chen et al., 2019). Besides, *Akkermansia muciniphila* showed prominent negative correlations with several differential metabolites such as Phenylacetylglycine, (±)11(12)-eet, 15(r),19(r)-hydroxy prostaglandin f2α, Butylparaben, Sedanolide, Medroxyprogesterone, Ethyl oleate, Diacetyl and Vanillin. Among these differential metabolites, Phenylacetylglycine was reported to be abnormally elevated in prostate and colon cancer and may be a candidate marker for prostate and colon cancer (Li Z. et al., 2021; Yang B. et al., 2021); Butylparaben may enhance the migration and invasion activity of human breast cancer cells *in vitro* (Khanna et al., 2014). Therefore, it can be speculated that the anti-hepatoma mechanism of XRZYBXD may be associated with the improvement of intestinal flora and microbial metabolites.

Dysregulation of intestinal flora can impair intestinal barrier function through a variety of mechanisms (Odenwald and Turner, 2017). Impaired intestinal barrier function leads to the translocation of a large number of intestinal harmful bacteria, endotoxins and harmful metabolites into the liver, causing liver inflammation and fibrosis. Continuous stimulation of liver inflammation causes excessive cell proliferation and resistance to death, thus causing the occurrence and progression of liver cancer (Schneider et al., 2022). This is consistent with our results, we found that following modeling, the ileum structure was disturbed, the villi were broken and the goblet cells were reduced and the mucosal epithelium of the ileum was necrotic and exfoliated, however, after treatment of XRZYBXD high dose, the pathological changes of the ileum structure, mucosa and goblet cells were restored. In addition, occluding, claudin-1 and ZO-1, which are important components of intestinal tight junction proteins, play an important role in repairing intestinal mucosa, maintaining mucosal epithelial mechanical barrier and permeability, and are reliable indicators for evaluating intestinal tight junction function (Kuo et al., 2021). In our study, compared with normal group, the expression of occluding, claudin-1 and ZO-1 in tumor model group were prominently reduced, but they were all restored after the intervention of XRZYBXD. Besides, spearman correlation analysis showed that the indicators of intestinal barrier function had close association with microflora. These results further suggested that XRZYBXD may have anti-hepatoma effect through restoring dysfunctional gut microbiota.

**FIGURE 8:**
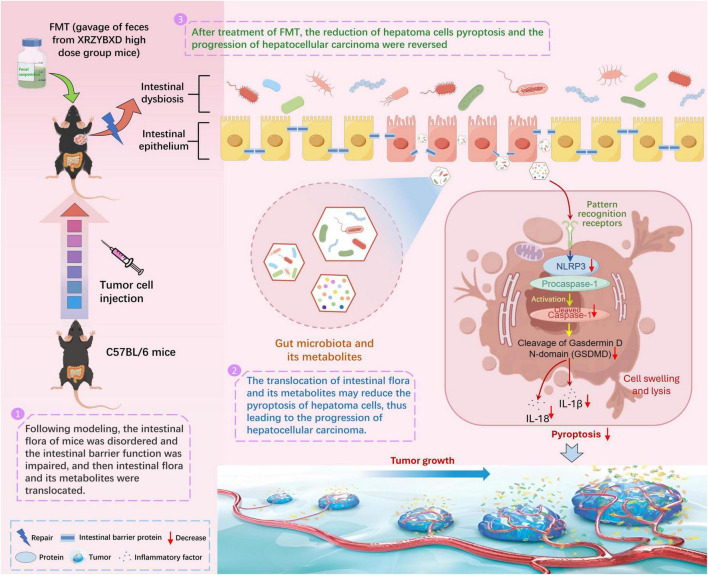
Mechanism diagram.

In order to further prove the role of gut microbiota in the anti-hepatoma effect of XRZYBXD, microbial manipulation experiments were conducted. We found that elimination of gut microbiota by antibiotics weakened the anti-hepatoma effect and the promotion effect on hepatoma cell pyroptosis for XRZYBXD. However, transplanting XRZYBXD-shaped intestinal flora showed a prominent anti-hepatoma effect and a promoting effect on hepatoma cell pyroptosis. These demonstrated that gut microbiota may mediate the anti-hepatoma and pro-pyroptosis efficacy of XRZYBXD (Figure 8).

Although this study validated that XRZYBXD may induce pyroptosis of hepatoma cells by regulating intestinal flora and metabolites, there are still some shortcomings. First of all, how gut microbiota and metabolites mediate the promoting effect of XRZYBXD on hepatoma cell pyroptosis should be further investigated. Secondly, the effect of specific metabolites of XRZYBXD on intestinal flora and metabolites, as well as the decomposition and metabolism effect of intestinal flora on specific components of XRZYBXD have not been uncovered. Third, the role of specific strains (such as *Akkermansia muciniphila*) in the anti-tumor and pro-pyroptosis effects of XRZYBXD for liver cancer also needs to be further clarified.

## 5 Conclusion

XRZYBXD promote pyroptosis of hepatoma cells via adjusting intestinal dysbiosis.

## Data Availability

The original contributions presented in this study are included in this article/Supplementary material, further inquiries can be directed to the corresponding authors.

## Footnotes

1
http://mpns.kew.org

## Supplementary material

Supplementary Figure 1 30 specific chemical metabolites of XRZYBXD were identified based on Q-Orbitrap high-resolution liquid mass spectrometry. **(A)** Negative and **(B)** positive ion map.

Supplementary Figure 2 **(A)** H&E staining of ileum (magnification × 200), the scale bar represents 50 μm. **(B–D)** The mRNA expressions of Muc2 in ileum tissues using Q-PCR. **(E)** Petal diagram of gut microbiota. **(F–N)** The protein expressions of IL-1β and IL-18 in tumor tissues based on WB. All data are presented as means ± SD (*n* = 5). ABX, antibiotics; FMT, fecal microbiota transplantation; Muc2, mucin 2; Q-PCR, quantitative real time polymerase chain reaction; WB, western blot.

## References

[B1] Aggarwal V. Sunder S. Verma S. R. (2022). Disease-associated dysbiosis and potential therapeutic role of Akkermansia muciniphila, a mucus degrading bacteria of gut microbiome. *Folia Microbiol (Praha).* 67 811–824. 10.1007/s12223-022-00973-6 35596115 PMC9122250

[B2] Bai Y. Bao X. Mu Q. Fang X. Zhu R. Liu C. (2021). Ginsenoside Rb1, salvianolic acid B and their combination modulate gut microbiota and improve glucolipid metabolism in high-fat diet induced obese mice. *PeerJ.* 9:e10598. 10.7717/peerj.10598 33604164 PMC7866888

[B3] Bu F. Zhang S. Duan Z. Ding Y. Chen T. Wang R. (2020). A critical review on the relationship of herbal medicine, Akkermansia muciniphila, and human health. *Biomed. Pharmacother.* 128:110352. 10.1016/j.biopha.2020.110352 32521456

[B4] Cani P. D. Depommier C. Derrien M. Everard A. de Vos W. M. (2022). Akkermansia muciniphila: paradigm for next-generation beneficial microorganisms. *Nat. Rev. Gastroenterol. Hepatol.* 19 625–637. 35641786 10.1038/s41575-022-00631-9

[B5] Che Y. Shi X. Zhong X. Zhang Y. Si R. Li Y. (2020). Resveratrol prevents liver damage in MCD-induced steatohepatitis mice by promoting SIGIRR gene transcription. *J. Nutr. Biochem.* 82:108400. 10.1016/j.jnutbio.2020.108400 32438122

[B6] Chen H. Zhao C. He R. Zhou M. Liu Y. Guo X. (2019). Danthron suppresses autophagy and sensitizes pancreatic cancer cells to doxorubicin. *Toxicol In Vitro.* 54 345–353. 10.1016/j.tiv.2018.10.019 30389604

[B7] Chen T. T. Du S. L. Wang S. J. Wu L. Yin L. (2022). Dahuang Zhechong pills inhibit liver cancer growth in a mouse model by reversing Treg/Th1 balance. *Chin. J. Nat. Med*. 20, 102–110. 35279237 10.1016/S1875-5364(22)60160-2

[B8] Chou Y. T. Hsu F. F. Hu D. Y. Chen Y. C. Hsu Y. H. Hsu J. T. (2018). Identification of danthron as an isoform-specific inhibitor of HEME OXYGENASE-1/cytochrome P450 reductase interaction with anti-tumor activity. *J. Biomed. Sci.* 25:6. 10.1186/s12929-018-0411-y 29361943 PMC5781335

[B9] Dai C. M. Jin S. Zhang J. Z. (2021). Effect of Dahuang Zhechong Pills combined with TACE on VEGF,MMP-2,TGF-β1 and immune function of patients with primary liver cancer (blood stasis and collaterals blocking type). *China J. Chin. Mater. Med*. 46. 10.19540/j.cnki.cjcmm.20200716.501 33645040

[B10] Hanahan D. (2022). Hallmarks of cancer: new dimensions. *Cancer Discov.* 12 31–46. 35022204 10.1158/2159-8290.CD-21-1059

[B11] Hisaka T. Sakai H. Sato T. Goto Y. Nomura Y. Fukutomi S. (2020). Quercetin suppresses proliferation of liver cancer cell lines in vitro. *Anticancer Res.* 40 4695–4700. 32727794 10.21873/anticanres.14469

[B12] Hu Q. Wu C. Yu J. Luo J. Peng X. (2022). Angelica sinensis polysaccharide improves rheumatoid arthritis by modifying the expression of intestinal Cldn5, Slit3 and Rgs18 through gut microbiota. *Int. J. Biol. Macromol.* 209 (Pt. A) 153–161. 10.1016/j.ijbiomac.2022.03.090 35318077

[B13] Jia W. Xie G. Jia W. (2018). Bile acid-microbiota crosstalk in gastrointestinal inflammation and carcinogenesis. *Nat. Rev. Gastroenterol. Hepatol.* 15 111–128. 29018272 10.1038/nrgastro.2017.119 PMC5899973

[B14] Jiang N. Song X. Peng Y. M. Wang W. N. Song Z. (2020). Association of disease condition with changes in intestinal flora, and plasma endotoxin and vascular endothelial growth factor levels in patients with liver cancer. *Eur. Rev. Med. Pharmacol. Sci.* 24 3605–3613. 10.26355/eurrev_202004_20822 32329835

[B15] Jin B. Zhang C. Geng Y. Liu M. (2020). Therapeutic effect of ginsenoside rd on experimental autoimmune encephalomyelitis model mice: regulation of inflammation and Treg/Th17 cell balance. *Mediat. Inflamm.* 2020:8827527. 10.1155/2020/8827527 33380901 PMC7762661

[B16] Khanna S. Dash P. R. Darbre P. D. (2014). Exposure to parabens at the concentration of maximal proliferative response increases migratory and invasive activity of human breast cancer cells in vitro. *J. Appl. Toxicol.* 34 1051–1059. 10.1002/jat.3003 24652746

[B17] Koh G. Y. Kane A. Lee K. Xu Q. Wu X. Roper J. (2018). Parabacteroides distasonis attenuates toll-like receptor 4 signaling and Akt activation and blocks colon tumor formation in high-fat diet-fed azoxymethane-treated mice. *Int. J. Cancer* 143 1797–1805. 10.1002/ijc.31559 29696632

[B18] Kuo W. T. Zuo L. Odenwald M. A. Madha S. Singh G. Gurniak C. B. (2021). The tight junction protein ZO-1 Is dispensable for barrier function but critical for effective mucosal repair. *Gastroenterology* 161 1924–1939. 10.1053/j.gastro.2021.08.047 34478742 PMC8605999

[B19] Li S. Liang T. Zhang Y. Huang K. Yang S. Lv H. (2021). Vitexin alleviates high-fat diet induced brain oxidative stress and inflammation via anti-oxidant, anti-inflammatory and gut microbiota modulating properties. *Free Radic. Biol. Med.* 171 332–344. 10.1016/j.freeradbiomed.2021.05.028 34029693

[B20] Li Y. Sheng L. Jena P. K. Gilbert M. C. Wan Y. Y. Mao H. (2022). Retinoic acid signaling is compromised in DSS-induced dysbiosis. *Nutrients* 14:2788. 10.3390/nu14142788 35889745 PMC9315703

[B21] Li Z. Deng X. Luo J. Lei Y. Jin X. Zhu J. (2021). Metabolomic comparison of patients with colorectal cancer at different anticancer treatment stages. *Front. Oncol.* 11:574318. 10.3389/fonc.2021.574318 35186705 PMC8855116

[B22] Liu Q. Li F. Zhuang Y. Xu J. Wang J. Mao X. (2019). Alteration in gut microbiota associated with hepatitis B and non-hepatitis virus related hepatocellular carcinoma. *Gut Pathog.* 11:1. 10.1186/s13099-018-0281-6 30675188 PMC6337822

[B23] Liu X. Tang S. Zhong H. Tong X. Jie Z. Ding Q. (2021). A genome-wide association study for gut metagenome in Chinese adults illuminates complex diseases. *Cell Discov.* 7:9. 10.1038/s41421-020-00239-w 33563976 PMC7873036

[B24] Liwei Liu J. X. Cai S. Li Y. Jiang Y. (2023). Study on the mechanism of Dahuang Zhechong Pills against the proliferation of liver cancer based on network pharmachology. *Northwest J. Pharm* 38

[B25] Ma J. Li J. Jin C. Yang J. Zheng C. Chen K. (2023). Association of gut microbiome and primary liver cancer: a two-sample Mendelian randomization and case-control study. *Liver Int.* 43 221–233. 10.1111/liv.15466 36300678

[B26] Newton K. Strasser A. Kayagaki N. Dixit V. M. (2024). Cell death. *Cell* 187 235–256. 38242081 10.1016/j.cell.2023.11.044

[B27] Odenwald M. A. Turner J. R. (2017). The intestinal epithelial barrier: a therapeutic target? *Nat. Rev. Gastroenterol. Hepatol.* 14 9–21. 10.1038/nrgastro.2016.169 27848962 PMC5554468

[B28] Rodrigues V. F. Elias-Oliveira J. Pereira I. S. Pereira J. A. Barbosa S. C. Machado M. S. G. (2022). Akkermansia muciniphila and gut immune system: a good friendship that attenuates inflammatory bowel disease, obesity, and diabetes. *Front. Immunol.* 13:934695. 10.3389/fimmu.2022.934695 35874661 PMC9300896

[B29] Sanjiwani M. I. D. Aryadi I. P. H. Semadi I. M. S. (2022). Review of literature on akkermansia muciniphila and its possible role in the etiopathogenesis and therapy of type 2 diabetes mellitus. *J. ASEAN Fed. Endocr. Soc.* 37 69–74. 10.15605/jafes.037.01.13 35800592 PMC9242659

[B30] Schneider K. M. Mohs A. Gui W. Galvez E. J. C. Candels L. S. Hoenicke L. (2022). Imbalanced gut microbiota fuels hepatocellular carcinoma development by shaping the hepatic inflammatory microenvironment. *Nat. Commun.* 13:3964. 10.1038/s41467-022-31312-5 35803930 PMC9270328

[B31] Schwabe R. F. Jobin C. (2013). The microbiome and cancer. *Nat. Rev. Cancer* 13 800–812. 24132111 10.1038/nrc3610 PMC3986062

[B32] Shabbir U. Rubab M. Daliri E. B. Chelliah R. Javed A. Oh D. H. (2021). Curcumin, quercetin, catechins and metabolic diseases: the role of gut microbiota. *Nutrients* 13:206. 10.3390/nu13010206 PMC7828240 33445760

[B33] Sung H. Ferlay J. Siegel R. L. Laversanne M. Soerjomataram I. Jemal A. (2021). Global Cancer Statistics 2020: GLOBOCAN estimates of incidence and mortality worldwide for 36 cancers in 185 countries. *CA Cancer J. Clin.* 71 209–249. 33538338 10.3322/caac.21660

[B34] Touchefeu Y. Duchalais E. Bruley des Varannes S. Alameddine J. Mirallie E. Matysiak-Budnik T. (2021). Concomitant decrease of double-positive lymphocyte population CD4CD8alphaalpha and Faecalibacterium prausnitzii in patients with colorectal cancer. *Eur. J. Gastroenterol. Hepatol.* 32 149–156. 10.1097/MEG.0000000000001842 32675782

[B35] Vogel A. Meyer T. Sapisochin G. Salem R. Saborowski A. (2022). Hepatocellular carcinoma. *Lancet* 400 1345–1362. 36084663 10.1016/S0140-6736(22)01200-4

[B36] Wang C. Z. Yu C. Wen X. D. Chen L. Zhang C. F. Calway T. (2016). American ginseng attenuates colitis-associated colon carcinogenesis in mice: impact on gut microbiota and metabolomics. *Cancer Prev. Res. (Phila).* 9 803–811. 10.1158/1940-6207.CAPR-15-0372 27443884 PMC5052115

[B37] Wang C. Ruan P. Zhao Y. Li X. Wang J. Wu X. (2017). Spermidine/spermine N1-acetyltransferase regulates cell growth and metastasis via AKT/β-catenin signaling pathways in hepatocellular and colorectal carcinoma cells. *Oncotarget* 8 1092–1109. 27901475 10.18632/oncotarget.13582 PMC5352037

[B38] Wang W. J. Chen D. Jiang M. Z. Xu B. Li X. W. Chu Y. (2018). Downregulation of gasdermin D promotes gastric cancer proliferation by regulating cell cycle-related proteins. *J. Dig. Dis.* 19 74–83. 10.1111/1751-2980.12576 29314754

[B39] Wei Q. Mu K. Li T. Zhang Y. Yang Z. Jia X. (2014). Deregulation of the NLRP3 inflammasome in hepatic parenchymal cells during liver cancer progression. *Lab. Invest.* 94 52–62. 10.1038/labinvest.2013.126 24166187

[B40] Williams T. M. Leeth R. A. Rothschild D. E. Coutermarsh-Ott S. L. McDaniel D. K. Simmons A. E. (2015). The NLRP1 inflammasome attenuates colitis and colitis-associated tumorigenesis. *J. Immunol.* 194 3369–3380. 25725098 10.4049/jimmunol.1402098 PMC4369420

[B41] Xiang K. Li C. X. Chen R. Zhao C. H. (2024). Genetically predicted gut microbiome and risk of oral cancer. *Cancer Causes Control.* 35 429–435. 37815646 10.1007/s10552-023-01800-0

[B42] Xu C. Lee S. K. Zhang D. Frenette P. S. (2020). The gut microbiome regulates psychological-stress-induced inflammation. *Immunity* 53 417–28e4. 32735844 10.1016/j.immuni.2020.06.025 PMC7461158

[B43] Yang B. Zhang C. Cheng S. Li G. Griebel J. Neuhaus J. (2021). Novel metabolic signatures of prostate cancer revealed by (1)H-NMR metabolomics of urine. *Diagnostics (Basel)* 11:149. 10.3390/diagnostics11020149 33498542 PMC7909529

[B44] Yang L. Yu S. Yang Y. Wu H. Zhang X. Lei Y. (2021). Berberine improves liver injury induced glucose and lipid metabolic disorders via alleviating ER stress of hepatocytes and modulating gut microbiota in mice. *Bioorg. Med. Chem.* 55:116598. 10.1016/j.bmc.2021.116598 34979291

[B45] Zeng Q. Zhang M. Wang R. (2024). Causal link between gut microbiome and schizophrenia: a Mendelian randomization study. *Psychiatr. Genet.* 34 43–53. 38441075 10.1097/YPG.0000000000000361

[B46] Zeng Z. Jiang W. Kan J. Zhang D. Li R. He F. (2023). Shentao Ruangan formula promotes apoptosis via the E2F2-p53 pathway in hepatocellular carcinoma. *Phytomedicine* 109:154565. 10.1016/j.phymed.2022.154565 36610125

[B47] Zhang J. Song J. Wu D. Wang J. Dong W. (2015). Hesperetin induces the apoptosis of hepatocellular carcinoma cells via mitochondrial pathway mediated by the increased intracellular reactive oxygen species, ATP and calcium. *Med. Oncol.* 32:101. 10.1007/s12032-015-0516-z 25737432

[B48] Zhang N. Wang Z. Lv J. Zhang S. Liu Y. Liu T. (2022). Characterization of gut microbiota and exploration of potential predictive model for hepatocellular carcinoma microvascular invasion. *Front. Med (Lausanne).* 9:836369. 10.3389/fmed.2022.836369 35372388 PMC8971959

[B49] Zhang X. Coker O. O. Chu E. S. Fu K. Lau H. C. H. Wang Y. X. (2021). Dietary cholesterol drives fatty liver-associated liver cancer by modulating gut microbiota and metabolites. *Gut* 70 761–774. 10.1136/gutjnl-2019-319664 32694178 PMC7948195

[B50] Zhao Z. Xiong S. Wang R. Li Y. Wang X. Wang Y. (2019). Peri-tumor fibroblasts promote tumorigenesis and metastasis of hepatocellular carcinoma via Interleukin6/STAT3 signaling pathway. *Cancer Manag. Res.* 11 2889–2901. 10.2147/CMAR.S192263 31118769 PMC6489558

[B51] Zhong W. Wu K. Long Z. Zhou X. Zhong C. Wang S. (2022). Gut dysbiosis promotes prostate cancer progression and docetaxel resistance via activating NF-κB-IL6-STAT3 axis. *Microbiome* 10:94. 10.1186/s40168-022-01289-w 35710492 PMC9202177

[B52] Zhou J. C. Zhang X. W. (2019). Akkermansia muciniphila: a promising target for the therapy of metabolic syndrome and related diseases. *Chin. J. Nat. Med.* 17 835–841. 10.1016/S1875-5364(19)30101-3 31831130

