# Supplementary figures and images for: Gut microbiota mediates the pro-pyroptosis effect of xierezhuyubuxu decoction in hepatocellular carcinoma
Source: Front Microbiol. 2025 Feb 19;15:1481111. doi: 10.3389/fmicb.2024.1481111 (PMC11880294; doi:10.3389/fmicb.2024.1481111)

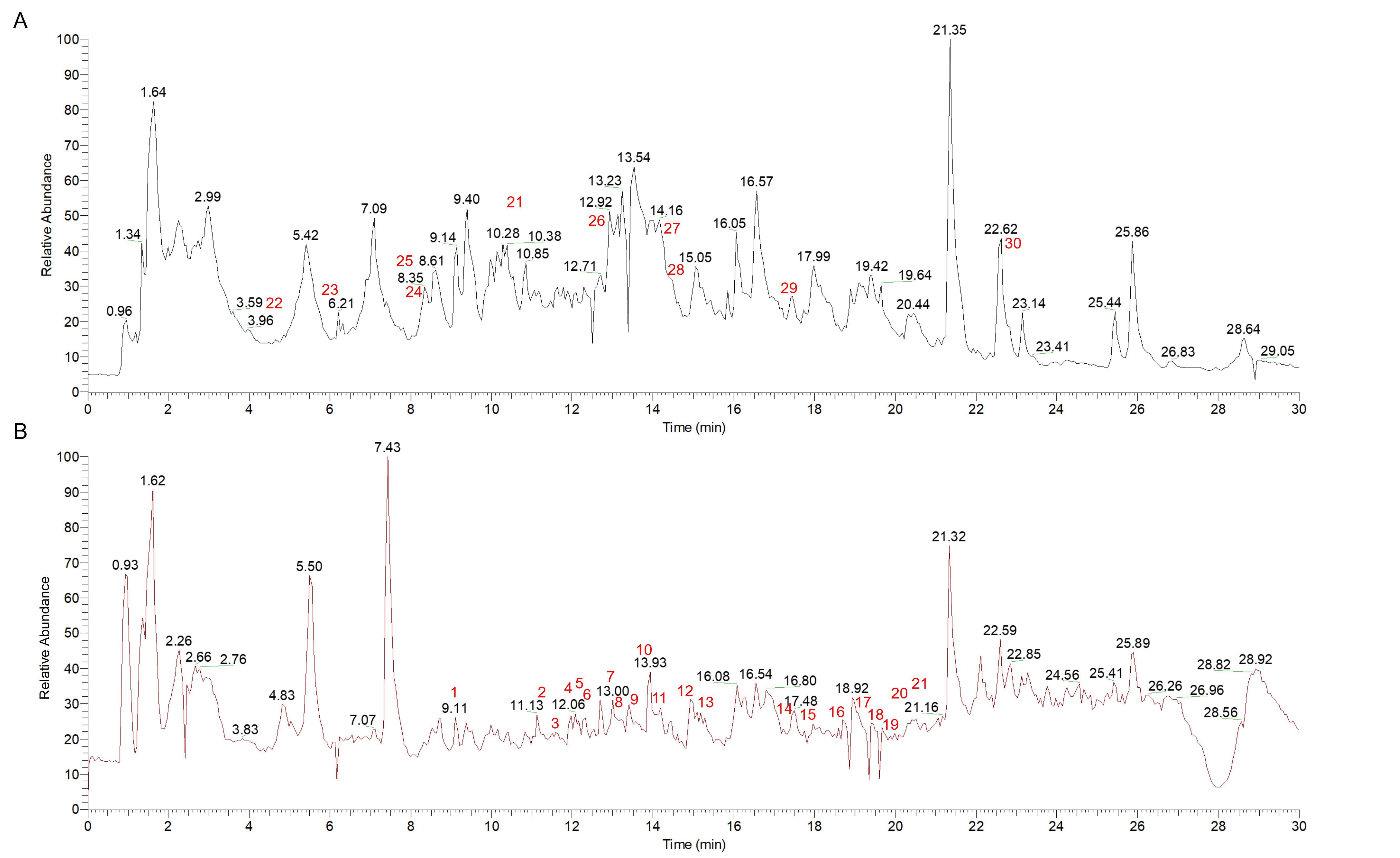

Supplement: Supplementary Figure 1 — 30 specific chemical metabolites of XRZYBXD were identified based on Q-Orbitrap high-resolution liquid mass spectrometry. (A) Negative and (B) positive ion map. [file Image_1.tif]

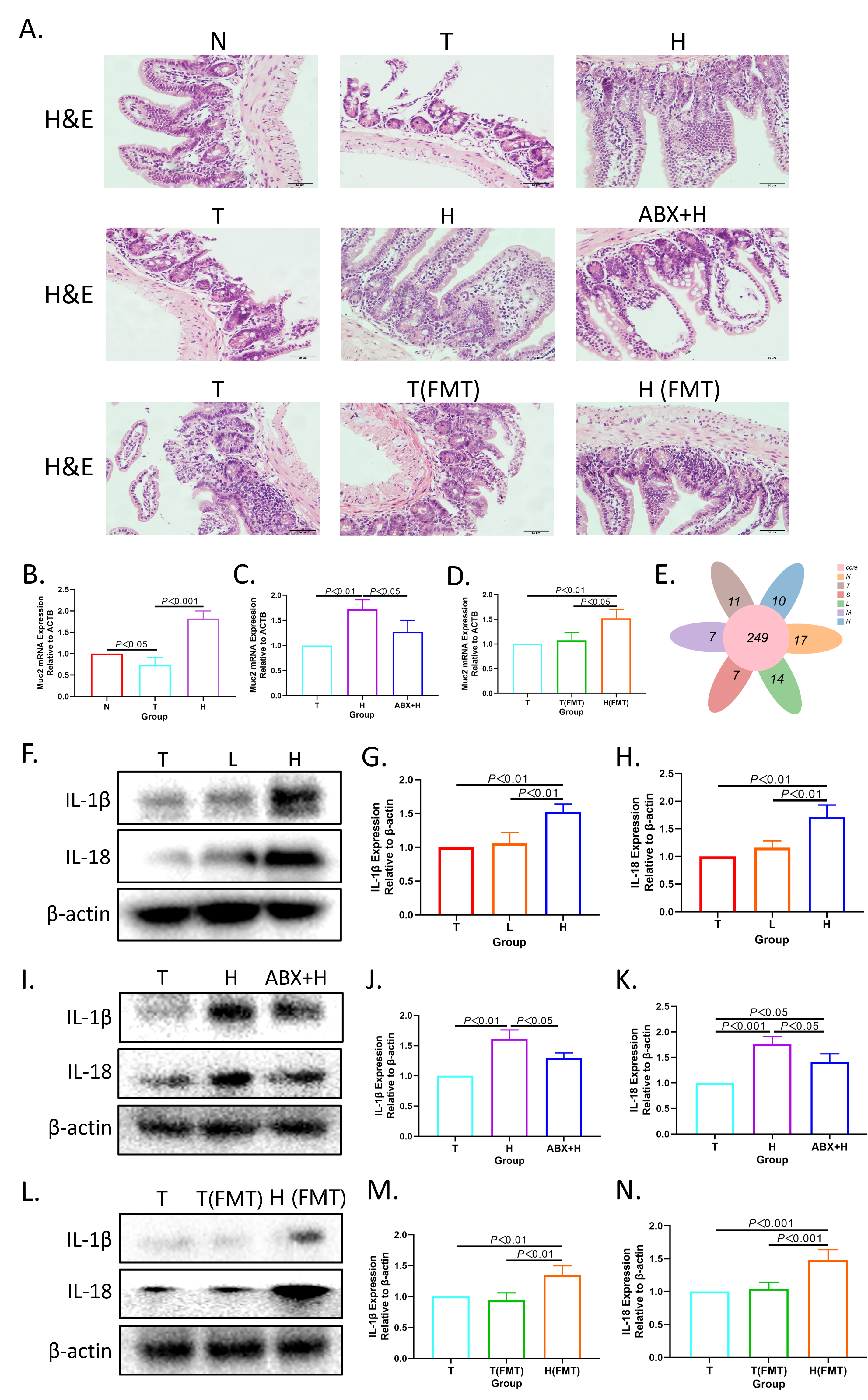

Supplement: Supplementary Figure 2 — (A) H&E staining of ileum (magnification × 200), the scale bar represents 50 μm. (B–D) The mRNA expressions of Muc2 in ileum tissues using Q-PCR. (E) Petal diagram of gut microbiota. (F–N) The protein expressions of IL-1β and IL-18 in tumor tissues based on WB. All data are presented as means ± SD (n = 5). ABX, antibiotics; FMT, fecal microbiota transplantation; Muc2, mucin 2; Q-PCR, quantitative real time polymerase chain reaction; WB, western blot. [file Image_2.tif]
